# Comparative Effects of Remimazolam and Propofol on Intraoperative Hypertension and Hypotension During Robot-Assisted Laparoscopic Gynecologic Surgery: A Retrospective Analysis

**DOI:** 10.3390/medicina61091721

**Published:** 2025-09-22

**Authors:** Jung Min Lee, Joohyun Lee, Se Hee Kang, Kangha Jung, Hyean Yeo, Young Joo

**Affiliations:** 1 Department of Anesthesiology and Pain Medicine, CHA Ilsan Medical Center, CHA University, Goyang-si 10414, Republic of Korea; vanilla228@chamc.co.kr (J.M.L.); sseya0316@chamc.co.kr (S.H.K.); kangha.jung11@chamc.co.kr (K.J.); hyean.yeo@chamc.co.kr (H.Y.); yjoosophia@gmail.com (Y.J.); 2 Department of Anesthesiology and Pain Medicine, College of Medicine, Kangwon National University, Chuncheon 24341, Republic of Korea

**Keywords:** remimazolam, propofol, general anesthesia, Trendelenburg position, pneumoperitoneum, hemodynamics, hypotension, hypertension, remifentanil, robotic surgical procedures

## Abstract

*Background and Objectives:* Remimazolam is a recently introduced benzodiazepine that has been increasingly adopted as an alternative to propofol. Although several trials have compared remimazolam with propofol, these studies have primarily focused on induction-related hypotension in non-gynecologic settings. To the best of our knowledge, both intraoperative hypertension and hypotension have not been systematically evaluated throughout the full anesthetic course in the specific physiologic context of robot-assisted laparoscopic gynecologic surgery performed in the steep Trendelenburg position with pneumoperitoneum. *Materials and Methods:* In this retrospective study, propensity score matching was performed to minimize selection bias. The demographic data of 694 patients, along with the incidence of intraoperative hypertension and hypotension, were collected through a review of medical records. *Results:* A total of 694 patients met the selection criteria, all of whom underwent total intravenous anesthesia (TIVA) using either remimazolam (*n* = 321) or propofol (*n* = 373). After propensity score matching, 317 pairs were analyzed. The incidence of intraoperative hypertension was higher in the remimazolam group (66.2% vs. 52.1%; *p* < 0.001), whereas hypotension was more frequent in the propofol group (12.0% vs. 5.4%; *p* = 0.003). *Conclusions:* TIVA with remimazolam was associated with a higher incidence of intraoperative hypertension compared to propofol, whereas propofol was more likely to cause hypotension in patients undergoing laparoscopic gynecologic surgery.

## 1. Introduction

Minimally invasive gynecologic surgery, including laparoscopic approaches, requires pneumoperitoneum and steep Trendelenburg positioning to optimize surgical exposure. These physiological conditions impose significant hemodynamic stress: while the Trendelenburg position may augment venous return and transiently increase cardiac output, pneumoperitoneum with CO_2_ insufflation elevates intra-abdominal pressure, potentially reducing cardiac preload and impairing cardiac function [1,2].

Propofol is widely used for total intravenous anesthesia (TIVA) due to its rapid onset and short duration of action [3]. However, hypotension and bradycardia are common during propofol-based TIVA [4], often compromising hemodynamic stability. Remimazolam, a novel benzodiazepine sedative rapidly metabolized by tissue esterases [5], has recently gained attention as an alternative TIVA agent. Emerging evidence suggests that remimazolam maintains adequate depth of anesthesia with a lower risk of post-induction hypotension, thereby contributing to more stable blood pressure control compared with propofol [6,7,8,9,10,11].

Although several studies have compared the hemodynamic effects of remimazolam and propofol, most have focused only on induction-related hypotension or were conducted in non-gynecologic procedures [6,9,10,11,12,13,14,15]. The intraoperative incidence of both hypertension and hypotension across the full duration of anesthesia, particularly in patients undergoing laparoscopic gynecologic surgery under Trendelenburg positioning, has not been comprehensively evaluated. To address this gap, we retrospectively compared intraoperative hypertension, hypotension, and associated vasoactive drug and opioid requirements between propofol- and remimazolam-based TIVA in laparoscopic gynecologic surgery.

## 2. Materials and Methods

**Figure 1:**
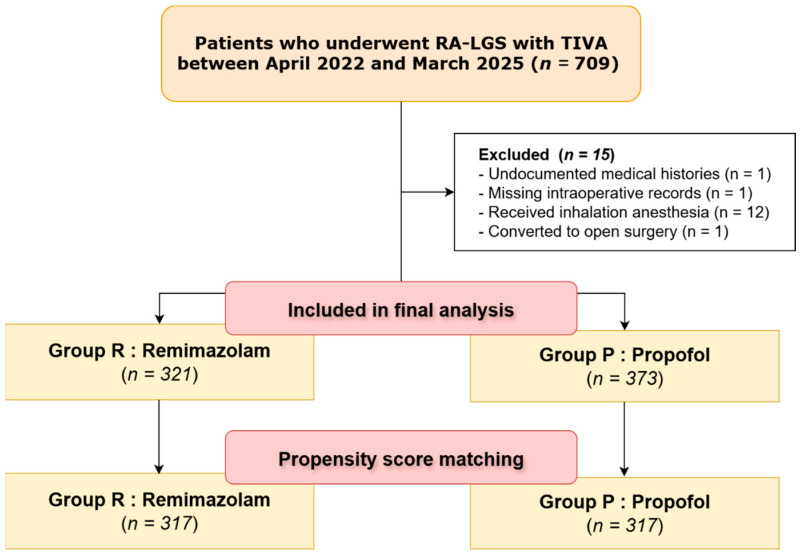
Study flow chart.

This retrospective study was approved by the Ethics Committee of CHA Ilsan Medical Center (approval number: ICHA 2024-06-004). We reviewed the medical records of patients who underwent robot-assisted laparoscopic gynecologic surgery (RA-LGS) with TIVA using either remimazolam (Group R) or propofol (Group P). Owing to the retrospective design, the requirement for written informed consent was waived, with patients given the opportunity to opt out of the study. In total, 709 patients who received the procedure with TIVA between April 2022 and March 2025 were initially identified. Patients were excluded if they had undocumented medical histories, had missing intraoperative records, received inhalation anesthetics, or underwent conversion to open surgery. After applying these criteria, 694 patients were included in the final analysis (Figure 1).

### 2.1. General Anesthesia

General anesthesia was induced in Group R with a continuous infusion of remimazolam at 6 or 12 mg/kg/h and remifentanil at 4 ng/mL of the effect site concentration (C*e*) via target-controlled infusion (TCI; Agilia®, Fresenius Kabi, Bad Homburg, Germany). In Group P, anesthesia was initiated with TCI of propofol at 4 mcg/mL of C*e* and remifentanil at 4 ng/mL. During anesthetic induction, a high infusion rate of crystalloid fluid was administered to facilitate rapid delivery of the anesthetic agent to the brain. After tracheal intubation was completed, the infusion rate was reduced to a level sufficient only to replace insensible water loss and maintained at this rate for the remainder of surgery. Maintenance of anesthesia was achieved using remimazolam (1–2 mg/kg/h) in Group R, and propofol (2.5–3.5 mcg/mL via TCI) in Group P with the goal of maintaining Patient State Index values (SedLine®, Masimo Corporation, Irvine, CA, USA) of 25–50. The C*e* of remifentanil in both groups was continuously titrated according to hemodynamic responses to surgical stimuli. Increases in blood pressure were interpreted as insufficient analgesia and prompted dose escalation, whereas decreases suggested relative opioid overdose and led to dose reduction. When blood pressure deviated beyond thresholds for more than 5 min despite a steady-state remifentanil C*e* or reached severe levels, vasoactive agents were administered. This strategy reflects standard intraoperative practice, aiming to maintain adequate analgesia while avoiding hypotension. Systolic and diastolic blood pressures (SBP and DBP) were recorded at 5 min intervals in handwritten anesthetic charts; mean arterial pressure (MAP) was not routinely documented. At the end of surgery, all patients received intravenous fentanyl or pethidine for immediate postoperative pain control, prophylactic intravenous ramosetron to prevent postoperative nausea and vomiting (PONV), and sugammadex to reverse the neuromuscular blockade induced by rocuronium. In cases where surgery ended earlier than anticipated and remimazolam tapering could not be adequately performed, flumazenil was administered at the discretion of the anesthesiologist to facilitate prompt emergence from anesthesia. Postoperative analgesia was managed using patient-controlled analgesia (PCA) with fentanyl.

### 2.2. Surgical Procedure

After induction of general anesthesia and completion of surgical preparation, skin incision was made. The time of incision was documented as a handwritten mark in the anesthetic chart. Within approximately 5 min after incision, patients were placed in the Trendelenburg position, with the angle maintained between 15° and 28° according to the surgeon’s preference. Abdominal insufflation with carbon dioxide was initiated at a pressure of 12 mmHg and a flow rate of 15 L/min, and this pneumoperitoneum was maintained throughout surgery. In some cases, intramyometrial vasopressin was injected to induce vasoconstriction of the myometrial vessels and reduce intraoperative bleeding.

### 2.3. Data Collection

Electronic medical and anesthesia records were reviewed to extract the following information. Preoperative data included age, weight, height, and past medical and medication histories, hemoglobin concentration, and ward SBP. Intraoperative data encompassed the diagnosis, type of procedure, degree of bed tilting, intraoperative vital signs, durations of surgery and anesthesia, use of vasopressin in the surgical field, intraoperative fluid intake and output, estimated blood loss (EBL) and red blood cell transfusion requirements, remifentanil dosage, and administration of medications to stabilize vital signs (e.g., ephedrine, phenylephrine, nicardipine, labetalol, esmolol, or atropine). Postoperative data included the numeric rating scale (NRS) score and vital signs in the postanesthesia care unit (PACU), PACU length of stay, total hospital length of stay, requirements for postoperative analgesia, the incidence and characteristics of postoperative pain, and incidence of PONV.

Intraoperative blood pressure values were obtained from handwritten anesthetic charts, where SBP and DBP were marked on a 10 mmHg scale, providing approximate rather than exact numerical values. As a result, MAP could not be reliably calculated and was therefore not collected. The data collection for both groups was performed using identical methods to ensure comparability.

To ensure data accuracy, two investigators independently reviewed these charts for the primary outcomes, yielding an inter-rater reliability of 0.93; any discrepancies were resolved by a third investigator. All other perioperative variables, including demographic characteristics, anesthetic drug dosages, intraoperative fluid balance, and postoperative outcomes, were retrieved from the electronic medical record system.

### 2.4. Outcomes

#### 2.4.1. Primary Outcomes

The primary outcomes of this study were the incidences of intraoperative hypertension and hypotension during anesthesia. Hypertension was defined as SBP ≥ 140 mmHg [16] and hypotension as SBP < 90 mmHg [17], in line with previous perioperative studies.

We further evaluated the specific features of intraoperative hypertension and hypotension through subgroup analyses. These included stratification by intraoperative phase, hemodynamic burden, and severity. For phase stratification, events were classified as occurring before incision, a period corresponding to anesthesia induction and preparation in the supine position with minimal noxious stimulation (other than tracheal intubation), or after incision, which encompassed the subsequent intraoperative period during which pneumoperitoneum and the Trendelenburg position were established and continuously maintained. Hemodynamic burden was defined as the proportion of intraoperative time with blood pressure outside the predefined thresholds. The proportion of intraoperative hypotension or hypertension was calculated as the number of time points at which SBP was below or above the predefined threshold divided by the total number of SBP measurements recorded during anesthesia. Severe cases were defined as SBP ≥ 160 mmHg for hypertension and SBP < 80 mmHg for hypotension, respectively [18].

#### 2.4.2. Secondary Outcomes

Secondary outcomes included variables that serve as indirect indicators of intraoperative hemodynamic responses: total remifentanil dose and the use of vasoactive agents to manage blood pressure (i.e., vasopressors, such as ephedrine or phenylephrine, and antihypertensives, such as nicardipine, esmolol, or labetalol).

These parameters were considered clinically relevant because anesthesiologists administer titrate opioid and vasoactive drugs in response to elevations or decreases in blood pressure during surgery, making them meaningful surrogate markers of hemodynamic control rather than simple confounders.

#### 2.4.3. Exploratory Outcomes

Exploratory outcomes included intraoperative hemorrhage-related values (e.g., EBL and RBC transfusion), postoperative recovery parameters (PACU systolic blood pressure, numeric rating scale, and length of stay), the need for rescue analgesics—used as a surrogate marker for postoperative pain—and the incidence of PONV, which were evaluated within 48 h following surgery. Rescue analgesic use was defined as the administration of additional analgesics (acetaminophen, ibuprofen, ketorolac, or tramadol) within 48 h postoperatively in patients who reported an NRS pain score ≥4 despite receiving standard analgesic regimens. PONV was defined as the occurrence of any of the following within 48 h of surgery: administration of rescue antiemetics, documented episodes of vomiting, or clamping of PCA due to nausea or vomiting.

#### 2.4.4. Statistical Analyses

Sporadic missing entries (<0.2%) were handled by listwise deletion, with negligible impact on the analyses. A post hoc power analysis was conducted using G*Power 3.1, predicated on the observed rates of hypertension (67.3% vs. 55.5%) and hypotension (5.3% vs. 12.6%) in this study. With α = 0.05 and the observed group sizes (*n* = 321 and *n* = 373), the attained power levels were 0.88 and 0.92, respectively.

As sensitivity analyses, intraoperative hypertension and hypotension were alternatively defined as (i) any single occurrence of blood pressure crossing the predefined threshold at least once (“once”) or sustained episodes defined by (ii) two or more consecutive 5 min recordings (“≥5 min”) and (iii) three or more consecutive 5 min recordings (“≥10 min”), respectively.

Statistical analyses were conducted using SPSS Statistics version 26 (IBM Corp., Armonk, NY, USA) and SigmaStat version 4.0 (Systat Software Inc., Palo Alto, CA, USA). The normality of continuous variables was assessed using the Shapiro–Wilk test. Two-tailed *p*-values < 0.05 were considered statistically significant. Data are presented as the mean ± standard deviation for normally distributed variables and as the median with interquartile ranges for non-normally distributed variables. Group comparisons were performed using Student’s *t*-test or the Mann–Whitney U test, as appropriate. Categorical variables were analyzed using the chi-square test or Fisher’s exact test.

To address potential confounding for matching groups, we performed propensity score matching (PSM). Propensity scores were estimated using logistic regression, including age, BMI, history of hypertension, history of thyroid disease, American Society of Anesthesiologists (ASA) physical status, SBP in ward, and surgeon identifier. Patients were matched 1:1 by nearest-neighbor matching without replacement and with a caliper of 0.2 the standard deviation (SD) of the logit of the propensity score. No missing values were present in the covariates used for propensity score estimation. Balance diagnostics confirmed that all covariates were well balanced after matching (all standardized mean differences < 0.1), and no patients were discarded due to a lack of common support.

For multivariable regression, covariates were selected if they showed statistical significance in univariate analyses (*p* < 0.05) or were considered clinically relevant potential confounders (e.g., preoperative SBP, history of hypertension) based on prior literature and expert consensus [19,20].

## 3. Results

Data from 694 patients were analyzed, with 321 patients in Group R and 373 in Group P. Among 694 patients, data on fluid output were missing in 18 cases (2.6%), and data on EBL were missing in 25 cases (3.6%) due to incomplete anesthesia records. Propensity score matching utilizing nearest-neighbor matching was conducted, resulting in 634 patients being paired into 317 pairs.

Table 1 presents the demographic characteristics of the two groups prior to and following matching. There were no significant differences between the two groups.

The primary outcomes—occurrences of intraoperative hypertension and hypotension—are summarized in Table 2. After matching, hypertension was significantly more frequent in Group R than in Group P (66.2% vs. 52.1%; *P* < 0.001), and the incidences of sustained hypertension lasting ≥5 and ≥10 min were consistently higher in Group R. The absolute risk differences for hypertension were 14.2% (95% CI: 6.6–21.8) for any occurrence, 11.0% (95% CI: 3.4–18.7) for ≥ 5 min, and 17.0% (95% CI: 10.0–24.1) for ≥10 min, respectively.

Hypotension was more frequent in Group P than in Group R (12.0% vs. 5.4%; *p* = 0.003). The incidence of hypotension lasting ≥5 min was higher in Group R, whereas there was no significant difference between the two groups in the incidence of hypotension lasting ≥10 min. The absolute risk differences for hypotension were −6.6% (95% CI: −11.0 to −2.3) for any occurrence and −3.5% (95% CI: –6.4 to −0.6) for ≥5 min, respectively.

We also analyzed the characteristics of intraoperative hypertension and hypotension events (Table 3). Of the 375 patients who developed hypertension (210 in Group R and 165 in Group P), the prevalence of hypertension occurring either prior to or following surgical incision was comparable between the two groups; nonetheless, the incidence of hypertension manifesting both before and after incision was significantly elevated in Group R (27.6% vs. 16.4%; *p* = 0.010). The incidence of severe hypertension with SBP ≥ 160 mmHg did not differ substantially between the groups; however, the proportion was greater in Group R (*p* < 0.001).

Conversely, among the 55 patients who exhibited hypotension (17 in Group R and 38 in Group P), no significant differences were seen between the groups regarding timepoint distribution or proportion. Severe hypotension with SBP below 80 mmHg was not observed in either group.

Group R required a higher total dosage of remifentanil and a less frequent application of pharmacological interventions to stabilize vital signs compared to Group P (Table 4).

Intraoperative exploratory variables, including pre-anesthesia SBP in the operating room, type of surgery, bed tilting angle for Trendelenburg position, the durations of both operation and anesthesia, the number of patients who received vasopressin during the procedure, and intraoperative fluid output, did not differ significantly between the two groups. However, Group R received a significantly greater volume of intraoperative fluids (Table 5).

Postoperative exploratory outcomes are also summarized in Table 5. Although values immediately prior to discharge from the PACU were significantly higher in Group R, there were no significant differences found in the PACU NRS scores, duration of stay in PACU, length of hospital stay, the number of patients receiving blood products within 48 h, or PONV. No significant difference was observed in the number of patients receiving rescue analgesics between Group R and P. Among the reasons for increased analgesic uses, the incidence of headaches considerably varied between the two groups both prior to (12.8% vs. 6.7%) and following matching (12.6% vs. 6.6%).

Table 6 presents the outcomes of univariate and multivariate logistic regression analyses on the occurrence of intraoperative hypertension. Remimazolam infusion (*p* = 0.001), pre-induction hypertension in the operating room (*p* < 0.001 for 140 ≤ SBP < 160 mmHg, *p* < 0.001 for SBP ≥ 160 mmHg, respectively), elevated bed tilting angle for Trendelenburg position (*p* = 0.019), and intraoperative fluid administration (*p* = 0.049) were substantially correlated with an augmented risk of intraoperative hypertension.

Conversely, intraoperative hypotension was significantly associated with older age (*p* = 0.019) and a medical history of hypertension (*p* = 0.015) (Table 7). Remimazolam infusion (*p* = 0.002) and pre-induction hypertension in the operating room (*p* < 0.001 for 140 ≤ SBP < 160 mmHg, *p* < 0.001 for SBP ≥ 160 mmHg, respectively) were found to be protective factors against intraoperative hypotension (Table 7).

## 4. Discussion

We compared the hemodynamic effects of remimazolam and propofol in patients undergoing robot-assisted laparoscopic gynecologic surgery (RA-LGS) in the Trendelenburg position. Our findings demonstrate that hypertension was more frequently associated with remimazolam, whereas propofol was linked to a higher incidence of hypotension. Given the retrospective observational design, these results should be interpreted as associative rather than causal. Nevertheless, when interpreted in the clinical context, this pattern may provide valuable insights into the hemodynamic profiles of the two agents.

Previous studies have evaluated the hemodynamic effects of remimazolam and propofol during TIVA [6,9,10,11,12,13,14,15], consistently reporting that both induction and maintenance with propofol are associated with a higher incidence of hypotensive episodes compared to remimazolam. The reported incidence of hypotension during anesthesia with remimazolam has ranged from 1.4% to 38.1%, whereas for propofol, it has ranged from 10.1% to 64.6%. These variations likely reflect differences in study conditions, including operative settings, inclusion criteria, patient comorbidities, and definitions of hypotension. Consistent with prior findings, our study demonstrated that hypotension occurred more frequently in frequently in propofol group. According to the subgroup analysis of patients with hypotension in this study, the proportion of intraoperative time below the SBP threshold did not differ between the groups, and no episodes of severe hypotension (SBP < 80 mmHg) were observed. Intraoperative hypotension appeared to be transient and was managed with increased administration of vasoactive agents (e.g., ephedrine or phenylephrine). Established risk factors for intraoperative hypotension include advanced age, pre-induction hypotension, and the use of antihypertensive medications [21]. Our findings align with these risk factors, as both age and a history of hypertension were significantly associated with hypotension in the propofol group.

Previous studies have consistently shown that remimazolam anesthesia provides stable hemodynamic profiles, often reflected in higher mean arterial pressure compared to other agents [9,12,13,14,22]. However, to date, no studies have reported remimazolam-associated hypertensive events. Our findings suggest that although remimazolam may contribute to the maintenance of blood pressure, it may also be associated with an increased incidence of intraoperative hypertension. In the subgroup analysis of patients who developed hypertension, the percentage of patients who experienced hypertension both before and after incision, as well as the overall proportion of measurements above the threshold, was higher in the remimazolam group. Hypertension occurred more frequently and repeatedly throughout the entire intraoperative period in the remimazolam group, despite increased infusion rates of remifentanil to attenuate blood pressure elevation.

The occurrence of hypertension did not significantly affect the intraoperative use of beta-blockers or calcium channel blockers to control hypertension, which may reflect a clinical tendency of physicians to initially increase remifentanil infusion to manage elevated blood pressure and reserve antihypertensive drugs as secondary options. Patients who received remimazolam infusion were administered higher doses of remifentanil. This finding is consistent with previous studies reporting a similar trend in the remimazolam group [6,9], which may be attributed to the more liberal intraoperative use of remifentanil in response to elevated blood pressure associated with remimazolam.

Uncontrolled intraoperative hypertension has been associated with adverse postoperative outcomes, including myocardial ischemia [23], stroke, intracranial hemorrhage, and acute kidney injury [24]. Additionally, it may increase the risk of postoperative delirium [25] and surgical site bleeding [26]. To mitigate these risks, anesthesiologists should carefully maintain intraoperative blood pressure close to the patient’s baseline. Within this clinical context, our study demonstrated that while remimazolam was associated with a higher overall incidence of intraoperative hypertension compared with propofol, the occurrence of severe hypertension did not differ between groups. This pattern likely reflects proactive intraoperative management, as anesthesiologists titrated opioids to maintain blood pressure within a target range. Supporting this interpretation, remifentanil consumption was significantly greater in the remimazolam group.

The hemodynamic patterns observed in this study—transient hypotension in the propofol group and repeated hypertension in the remimazolam group—appear to be also influenced by the combined effects of CO_2_ pneumoperitoneum and the Trendelenburg position, both essential for performing RA-LGS. Previous research has suggested that this intraoperative setting may elevate mean arterial pressure and central venous pressure due to increased hydrostatic pressure from the tilted position, augmented cardiac output, elevated systemic vascular resistance [27], and increased stroke volume [28]. Anesthesia with propofol may attenuate these hypertensive effects, whereas remimazolam, which has minimal impact on hemodynamic parameters, appears to lead to continued elevation of blood pressure. In addition to remimazolam infusion, intraoperative hypertension was also associated with elevated blood pressure recorded immediately prior to induction, higher tilt angle of surgical table, and intraoperative fluid input. Although a history of hypertension has previously been identified as a risk factor for intraoperative hypertension [29], it was not found to increase the risk significantly in our study.

Notably, intraoperative fluid input differed between groups, with the remimazolam group receiving approximately 150 mL more on average. This likely reflects the routine practice of maintaining a high-rate infusion during induction, and remimazolam’s slower onset may have prolonged this period of rapid administration [30]. Although 150 mL is small relative to the total intraoperative fluid volume, a weak yet statistically significant association with intraoperative hypertension was observed, suggesting that fluid management—particularly in patients with low circulating volume—can substantially influence hemodynamic outcomes. This imbalance may have contributed to the lower incidence of hypotension and higher incidence of hypertension in the remimazolam group.

Propofol is well known for its antiemetic properties [31], and remimazolam has also been associated with a reduced incidence of PONV compared to inhalational anesthetics [32]. However, findings regarding the relative superiority of propofol over remimazolam in preventing PONV remain inconsistent. Although some studies have reported a lower incidence of PONV with propofol [11,33], others have found no significant difference between the two agents [30,32,34]. In the present study, despite the presence of several well-established risk factors for PONV, such as female sex, gynecologic surgery, laparoscopic approach, and postoperative opioid use [35], both intravenous anesthetics have demonstrated comparable antiemetic effects.

In our study, the incidence of postoperative headache was higher in the remimazolam group compared with the propofol group (12.8% vs. 6.7%). The mechanisms underlying this observation remain unclear, but several hypotheses can be considered. Propofol has been reported to reduce the risk of postoperative headache [36,37], and its antimigraine properties have also been noted outside the surgical setting, with trials exploring its use for acute migraine management [38]. By contrast, remimazolam lacks such protective effects; as a benzodiazepine, it may even promote cerebral vasodilation, leading to alterations in intracranial vascular tone that could precipitate headache [39,40]. Furthermore, remimazolam is often reversed with flumazenil, and headache is a reported side effect of flumazenil [41,42]. In our cohort, 4 out of 26 patients (15.4%) who received flumazenil developed postoperative headache, suggesting that reversal could have contributed, at least in part, to the higher incidence observed in the remimazolam group.

This study has several limitations. First, the analysis was restricted to elective gynecologic surgery, involving mainly relatively healthy, middle-aged women. While this homogeneity enhanced internal validity, it inevitably limits generalizability to other surgical populations, particularly elderly patients, men, or those with significant cardiovascular comorbidities. Second, despite efforts to adjust for confounders through propensity score matching and multivariable analyses, the retrospective design inherently carries the risk of unmeasured confounding. In particular, provider-level clustering could not be modeled because anesthesiologist identifiers were not consistently recorded in our retrospective dataset. However, intravenous anesthetic management at our institution follows a uniform consensus protocol, which likely minimized inter-provider variability. Thus, while provider-level clustering effects cannot be entirely excluded, they are expected to have been negligible in this study. Third, outcome definitions were constrained by the coarse granularity of the anesthetic records. Blood pressure was documented only at 5 min intervals using approximate values, which precluded the use of MAP or baseline-relative thresholds and required reliance on SBP with absolute cut-offs. Consequently, continuous indices such as time-weighted averages or area under the curve could not be computed. We attempted to partially address this limitation by analyzing the proportion of intraoperative time outside predefined thresholds, but a precise time-based assessment was not feasible. Fourth, as remifentanil was dynamically titrated according to intraoperative blood pressure, the observed differences may have been partly mediated by this adjustment rather than solely reflecting the direct pharmacologic effects of the anesthetics. Fifth, remimazolam, as a novel agent without a reliable TCI model comparable to propofol, was administered via fixed-rate infusion, resulting in a difference in drug delivery methods between groups that may represent a potential confounder. Finally, our study demonstrated a relatively high incidence of PONV despite multimodal prophylaxis, potentially underestimating the relative difference between anesthetics. This likely reflects the rigorous definition of PONV applied, and our definition did not account for severity, suggesting that further studies incorporating standardized severity grading are warranted.

Despite these limitations, our study highlights the tendency toward intraoperative hypertension with remimazolam and provides foundational evidence for future investigations. As this was a retrospective observational study, the findings should be interpreted as associative rather than causal, underscoring the need for prospective validation, which can better control for potential confounding factors and overcome the above limitations.

## 5. Conclusions

TIVA with remimazolam was associated with a higher incidence of intraoperative hypertension compared to propofol, whereas propofol was more frequently linked to hypotension during laparoscopic surgery performed in the Trendelenburg position. Given the inherent limitations of a retrospective design and the possibility of residual confounding despite statistical adjustment, prospective studies are warranted to validate these findings.

## Figures and Tables

**Table 1:** Baseline characteristics of patients in the remimazolam and propofol groups before and after propensity score matching.

Variable	Before Matching			After Matching		
	Remimazolam (*n* = 321)	Propofol (*n* = 373)	*p* Value	Remimazolam (*n* = 317)	Propofol (*n* = 317)	*p* Value
Age (years)	41 (35–46)	42 (36–46)	0.543	41 (35–46)	42 (36.5–46)	0.393
Height (cm)	162 (158–165)	162 (159–166)	0.810	162 (158–165)	162 (158–166)	0.976
Weight (kg)	58 (53–65)	59 (53–65.6)	0.449	58.1 (53–65)	59 (53–65.1)	0.566
BMI (kg/m^2^)			0.1			0.253
underweight	27 (8.4)	15 (4)		26 (8.1)	14 (4.4)	
normal	157 (48.9)	197 (52.8)		155 (48.9)	163 (51.4)	
overweight	71 (22.1)	88 (23.6)		66 (20.8)	64 (20.2)	
obese	66 (20.6)	73 (18.6)		70 (22.1)	76 (24.0)	
History of HTN	23 (7.2)	26 (7)	0.961	23 (7.3)	22 (6.9)	0.877
Thyroid disease	17 (5.3)	19 (5.1)	0.959	16 (5.0)	11 (3.5)	0.325
ASA-PS (I/II)	8/313	12/361	0.498	8/309	7/310	0.794
Hb (mg/dl)	12.7 (11.9–13.5)	12.8 (11.8–13.5)	0.705	12.7 (11.8–13.5)	12.8 (11.8–13.5)	0.779
Ward SBP (mmHg) *	119 (110–130)	119 (110–128)	0.840	120 (110–130)	119 (110–128)	0.785

Values are presented as number (%) or median (interquartile range). BMI: body mass index (underweight: BMI < 18.5, normal: 18.5 ≤ BMI < 23, overweight: 23 ≤ BMI < 25, obese: BMI ≥ 25, based on WHO Asia-Pacific guidelines); HTN: hypertension; ASA-PS: American Society of Anesthesiologists physical status, presenting as number of patients in class I/class II; Hb: preoperative hemoglobin; SBP: systolic blood pressure. * SBP measured on the day before surgery during preoperative ward admission.

**Table 2:** Primary outcomes: incidence of intraoperative hypertension and hypotension before and after propensity score matching in the remimazolam and propofol groups.

Events	Before Matching			After Matching			
	Remimazolam (*n* = 321)	Propofol (*n* = 373)	*p* Value	Remimazolam (*n* = 317)	Propofol (*n* = 317)	*p* Value	ARD (95% CIs)
Hypertension							
once	216 (67.3)	207 (55.5)	0.002 *	210 (66.2)	165 (52.1)	<0.001 *	14.2 (6.6–21.8)
≥5 min	159 (49.5)	147 (39.4)	0.009 *	158 (49.8)	123 (38.8)	0.005 *	11.0 (3.4–18.7)
≥10 min	124 (38.6)	83 (22.3)	<0.001 *	124 (39.1)	70 (22.1)	<0.001 *	17.0 (10.0–24.1)
Hypotension							
once	17 (5.3)	47 (12.6)	0.001 *	17 (5.4)	38 (12.0)	0.003 *	−6.6 (−11.0–−2.3)
≥5 min	6 (1.9)	17 (4.6)	0.049 *	6 (1.9)	17 (5.4)	0.019 *	−3.5 (−6.4–−0.6)
≥10 min	2 (0.6)	6 (1.6)	0.225	2 (0.6)	5 (1.6)	0.254	–

Values are presented as number (%). Hypertension and hypotension indicate any occurrence of systolic blood pressure >140 mmHg or <90 mmHg, respectively, during general anesthesia. ARD: absolute risk difference; CI: confidence interval. * *p* < 0.05.

**Table 3:** Subgroup analysis after propensity score matching: features of intraoperative hypertension and hypotension in the remimazolam and propofol groups.

Stratification	Hypertension			Hypotension		
	Remimazolam (*n* = 210)	Propofol (*n* = 165)	*p* Value	Remimazolam (*n* = 17)	Propofol (*n* = 38)	*p* Value
Phases						
before incision	4 (1.9)	7 (4.2)	0.183	13 (76.5)	30 (78.9)	0.837
during procedure	148 (70.5)	131 (79.4)	0.050	4 (23.5)	6 (15.8)	0.492
both phases	58 (27.6)	27 (16.4)	0.010 *	0 (0.0)	2 (5.3)	0.335
Hemodynamic burden	0.20 (0.07–0.48)	0.13 (0.07–0.28)	<0.001 *	0.03 (0.03–0.07)	0.03 (0.03–0.07)	0.911
Severe cases	69 (32.9)	49 (29.7)	0.513	0	0	N/A

Values are presented as number (%) or median (interquartile range). N/A: not applicable. * *p* < 0.05.

**Table 4:** Secondary outcomes: remifentanil dose and vasoactive agent use before and after propensity score matching in the remimazolam and propofol groups.

Variable	Before Matching			After Matching		
	Remimazolam (*n* = 321)	Propofol (*n* = 373)	*p* Value	Remimazolam (*n* = 317)	Propofol (*n* = 317)	*p* Value
Remifentanil (mcg/kg/min)	0.27 (0.13–0.45)	0.05 (0.04–0.06)	<0.001 *	0.27 (0.12–0.45)	0.05 (0.04–0.062)	<0.001 *
Vasoactive agents						
vasopressors	34 (10.6)	69 (18.5)	0.003 *	34 (10.7)	66 (20.8)	<0.001 *
antihypertensives	63 (19.6)	82 (22.0)	0.504	62 (19.6)	66 (20.8)	0.692

Values are presented as a number (%) or median (interquartile range). * *p* < 0.05.

**Table 5:** Intra- and post-operative characteristics including exploratory outcomes: data before and after propensity score matching in the remimazolam and propofol groups.

Variable	Before Matching			After Matching		
	Remimazolam (*n* = 321)	Propofol (*n* = 373)	*p* Value	Remimazolam (*n* = 317)	Propofol (*n* = 317)	*p* Value
Intraoperative						
OR SBP (mmHg) ^†^	135 (123–150)	135 (123–147)	0.685	135 (124–150)	136 (123–147)	0.680
Surgery type			0.576			0.430
myomectomy	136 (42.4)	145 (38.9)		136 (42.9)	124 (39.1)	
TLH ± BSO	96 (29.9)	134 (35.9)		95 (30.0)	118 (37.2)	
combination	47 (14.6)	49 (13.1)		46 (14.5)	40 (12.6)	
OC	38 (11.8)	40 (10.7)		36 (11.4)	31 (9.8)	
staging	4 (1.2)	5 (1.3)		4 (1.3)	4(1.3)	
Tilting angle			0.457			0.253
15°	87 (27.1)	113 (30.3)		85 (26.8)	100 (31.5)	
20°	163 (50.8)	190 (50.9)		161 (50.8)	160 (50.5)	
23–28°	71 (22.1)	70 (18.8)		71 (22.4)	57 (18.0)	
OP duration (min)	120 (90–155)	120 (90–145)	0.547	120 (95–157)	120 (90–145)	0.412
ANS duration (min)	145 (115–182)	145 (115–170)	0.432	145 (118–184.5)	145 (115–170)	0.344
Vasopressin ^‡^	173 (53.9)	188 (50.4)	0.400	173 (54.6)	159 (50.2)	0.266
Input (mL)	950 (750–1175)	800 (550–1100)	<0.001 *	950 (750–1200)	800 (550–1150)	<0.001 *
Output (mL)	500 (300–700)	450 (319–700)	0.589	500 (340–700)	480 (330–700)	0.587
EBL (mL)	200 (100–350)	200 (100–375)	0.850	200 (100–350)	200 (100–400)	0.947
Atropine	1 (0.3)	7 (1.9)	0.117	1 (0.3)	6 (1.9)	0.057
Flumazenil	26 (8.1)	N/A	N/A	25 (7.89)	N/A	N/A
Postoperative						
RBC Transfusion	10 (3.1)	16 (4.3)	0.541	10 (3.2)	14 (4.4)	0.405
PACU SBP (mmHg)	125 (116–138)	122 (113–131)	<0.001 *	125 (116–138)	122 (113–132)	<0.001 *
NRS in PACU	2 (1–2)	2 (1–2)	0.601	2 (1–2)	2 (1–2)	0.637
PACU LOS (min)	28 (28–33)	28 (28–33)	0.223	28 (28–33)	28 (28–33)	0.544
Postoperative pain	160 (49.8)	167 (44.8)	0.208	158 (49.8)	144 (45.4)	0.266
MED of opioid (mg/kg)	1.41 ± 0.23	1.40 ± 0.21	0.179	1.41 ± 0.23	1.39 ± 0.20	0.467
characteristics						
OP site pain	137 (42.7)	146 (39.1)	0.385	135 (42.6)	127 (40.1)	0.519
headache	41 (12.8)	25 (6.7)	0.010 *	40 (12.6)	21 (6.6)	0.010 *
distension pain	9 (2.8)	11 (2.9)	0.910	8 (2.5)	10 (3.2)	0.633
PONV	163 (50.8)	206 (55.2)	0.208	162 (51.1)	177 (55.8)	0.232
Hospitalization LOS (h)	90 (89–92)	90 (88–92)	0.881	90 (89–92)	90 (88–92)	0.909

Values are presented as a number (%) or median (interquartile range). * *p* < 0.05. OR: operative room; SBP: systolic blood pressure; TLH: total laparoscopic hysterectomy; BSO: bilateral salphingo-oophorectomy; OC: ovary cystectomy, including right, left or both ovary; N/A: not applicable; MED: morphine equivalent dose; OP: operation; ANS: anesthesia; EBL: estimated blood loss. ^†^ SBP measured in the OR prior to anesthetic induction. ^‡^ Intramyometrial injection in surgical field.

**Table 6:** Univariate and multivariate logistic regression analysis of variables associated with intraoperative hypertension.

Variables	Univariate		Multivariate	
	OR (95% CIs)	*p* Value	OR (95% CIs)	*p* Value
Remimazolam vs. propofol	1.808 (1.312–2.491)	<0.001 *	1.949 (1.333–2.850)	0.001 *
Age	1.041 (1.017–1.65)	0.001 *	1.018 (0.988–1.049)	0.246
BMI				
Underweight	0.788 (0.408–1.522)	0.477	0.662 (0.318–1.380)	0.271
Overweight	1.265 (0.837–1.912)	0.265	0.992 (0.610–1.613)	0.974
Obese	2.565 (1.663–3.955)	<0.001 *	1.604 (0.963–2.670)	0.069
HTN history	1.986 (1.006–3.923)	0.048 *	0.415 (0.172–1.001)	0.050
Ward SBP ≥ 140 ^†^	11.174 (2.646–47.182)	0.001 *	2.383 (0.500–1.361)	0.276
OR SBP ^‡^				
140 ≤ SBP < 160	3.768 (2.553–5.563)	<0.001 *	4.335 (2.747–6.841)	<0.001 *
160 ≤ SBP	19.671 (7.021–55.111)	<0.001 *	18.251 (5.710–58.337)	<0.001 *
Tilting angle ^§^				
20°	1.545 (1.072–2.228)	0.020 *	1.543 (0.992–2.402)	0.055
23–28°	1.633 (1.030–2.588)	0.037 *	1.919 (1.113–3.311)	0.019 *
Vasopressin ^∥^	0.990 (0.721–1.360)	0.952	1.474 (0.977–2.226)	0.065
Input	1.001 (1.001–1.001)	<0.001 *	1.001 (1.000–1.001)	0.049 *
Output	1.001 (1.001–1.002)	<0.001 *	1.000 (0.999–1.001)	0.731

Model significance: *p* < 0.001, Nagelkerke R^2^; 0.289. OR: odds ratio; CI: confidence interval; BMI: body mass index (underweight: BMI < 18.5, normal: 18.5 ≤ BMI < 23, overweight: 23 ≤ BMI < 25, obese: BMI ≥ 25, based on WHO Asia-Pacific guidelines); HTN: hypertension; OR: operative room; SBP: systolic blood pressure. * *p* < 0.05. ^†^ SBP measured on the day before surgery during preoperative ward admission. ^‡^ SBP measured in the OR prior to anesthetic induction. ^§^ 15° was used as the reference. ^∥^ Intramyometrial injection in surgical field.

**Table 7:** Univariate and multivariate logistic regression analysis of variables associated with intraoperative hypotension.

Variables	Univariate		Multivariate	
	OR (95% CIs)	*p* Value	OR (95% CIs)	*p* Value
Remimazolam vs. propofol	0.416 (0.230–0.754)	0.004 *	0.353 (0.183–0.680)	0.002 *
Age	1.039 (0.998–1.082)	0.060	1.063 (1.010-1.118)	0.019 *
BMI				
Underweight	1.151 (0.382–3.469)	0.803	1.573 (0.492–5.034)	0.445
Overweight	1.053 (0.518–2.141)	0.886	1.099 (0.511–2.366)	0.809
Obese	0.844 (0.408–1.745)	0.647	1.067 (0.469–2.428)	0.876
HTN history	1.695 (0.684–4.203)	0.254	4.549 (1.347–15.364)	0.015 *
Ward SBP ≥ 140 ^†^	0.000 (0.000–0.000)	0.998		
OR SBP ^‡^				
140 ≤ SBP < 160	0.324 (0.149–0.700)	0.004 *	0.182 (0.071–0.467)	<0.001 *
160 ≤ SBP	0.108 (0.015–0.797)	0.029 *	0.041 (0.005–0.361)	<0.001 *
Tilting angle ^§^				
20°	0.572 (0.309–1.058)	0.075	0.550 (0.284–1.065)	0.076
23–28°	0.628 (0.287–1.375)	0.245	0.639 (0.277–1.473)	0.293
Vasopressin ^∥^	0.866 (0.498–1.506)	0.611	1.166 (0.609–2.230)	0.644
Input	0.999 (0.999–1.000)	0.069	1.000 (0.999–1.001)	0.518
Output	0.999 (0.998–1.000)	0.104	1.000 (0.998–1.001)	0.708

Model significance: *p* < 0.001, Nagelkerke R^2^; 0.174. OR: odds ratio; CI: confidence interval; BMI: body mass index (underweight: BMI < 18.5, normal: 18.5 ≤ BMI < 23, overweight: 23 ≤ BMI < 25, obese: BMI ≥ 25, based on WHO Asia-Pacific guidelines); HTN: hypertension; OR: operative room; SBP: systolic blood pressure. * *p* < 0.05. ^†^ SBP measured on the day before surgery during preoperative ward admission. ^‡^ SBP measured in the OR prior to anesthetic induction. § 15° was used as the reference. ^∥^ Intramyometrial injection in surgical field.

## Data Availability

Original contributions presented in this study are included in the article. Further inquiries can be directed to the corresponding author.

## Abbreviations

The following abbreviations are used in this manuscript:

TIVA	Total intravenous anesthesia
RA-LGS	Robot-assisted laparoscopic gynecologic surgery
TCI	Target-controlled infusion
PONV	Postoperative nausea and vomiting
PCA	Patient-controlled analgesia
NRS	Numeric rating scale
PACU	Postanesthesia care unit
SBP	Systolic blood pressure
